# Supplementary material for: Long-lived hot-carrier light emission and large blue shift in formamidinium tin triiodide perovskites
Source: Nat Commun. 2018 Jan 16;9:243. doi: 10.1038/s41467-017-02684-w (PMC5770436; doi:10.1038/s41467-017-02684-w)
Supplement: Supplementary file 1 — Supplementary Information [file 41467_2017_2684_MOESM1_ESM.pdf]

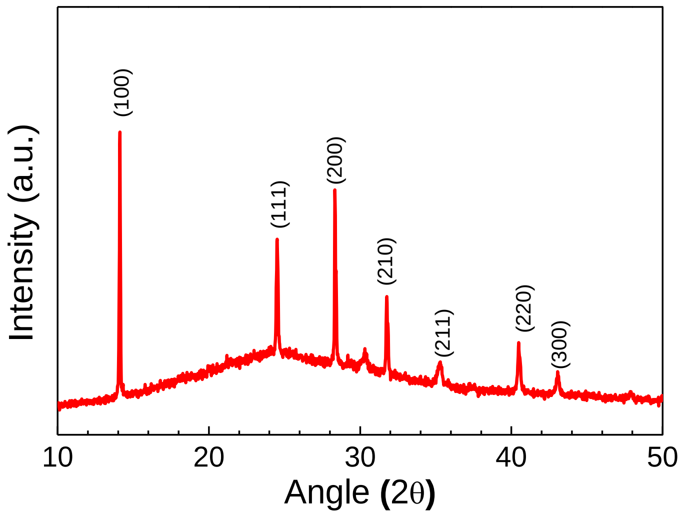

Supplementary Figure 1. Room temperature XRD pattern of the $FASnI_3$ thin film. The diffraction peaks are consistent with the structure reported in previous studies.[1]

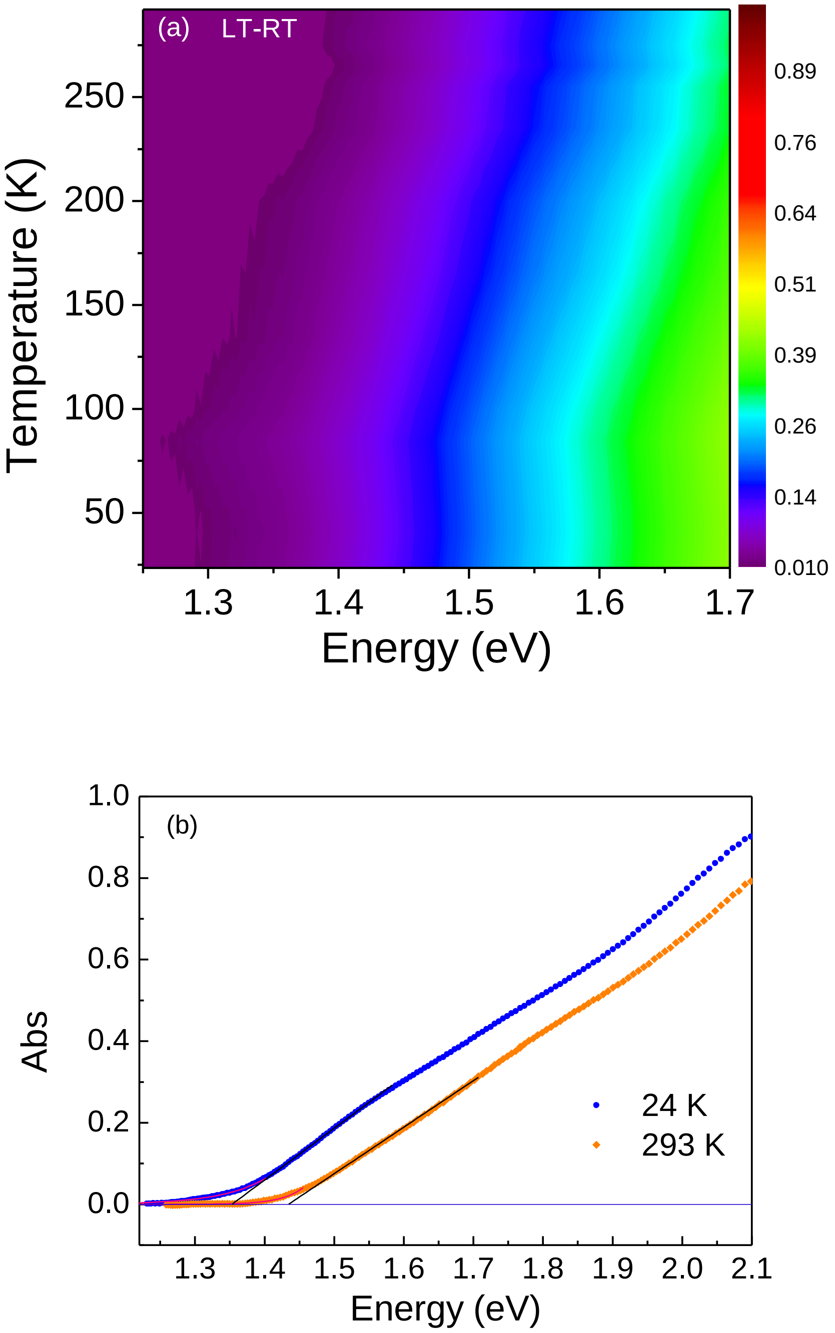

Supplementary Figure 2. (a) Contour plot of temperature dependent absorption spectra. (b) Absorption spectra of the $FASnI_3$ thin film sample at 24 K and 293 K. The energy dependence of the absorption coefficient of the Urbach tail is fitted by $\alpha(\hbar\omega) = \alpha(\hbar\omega_0)\exp[(\hbar\omega - \hbar\omega_0)/E_u]$, where $\hbar\omega_0$ is a parameter that defines the transition from the modified square-root law to an exponential dependence, E_u is the Urbach energy, a parameter related to the width of the tail.

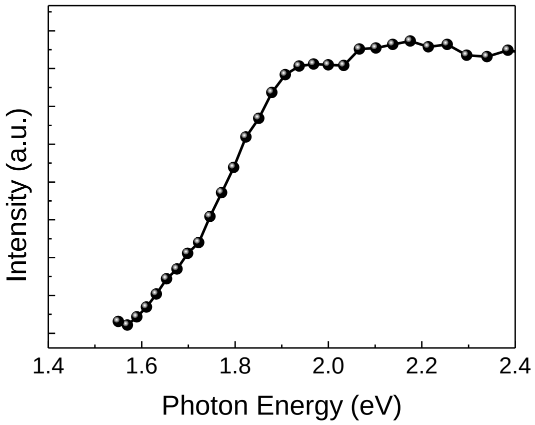

Supplementary Figure 3. Photoluminescence excitation (PLE) spectrum of $FASnI_3$ thin film (detection: 1.4 eV).

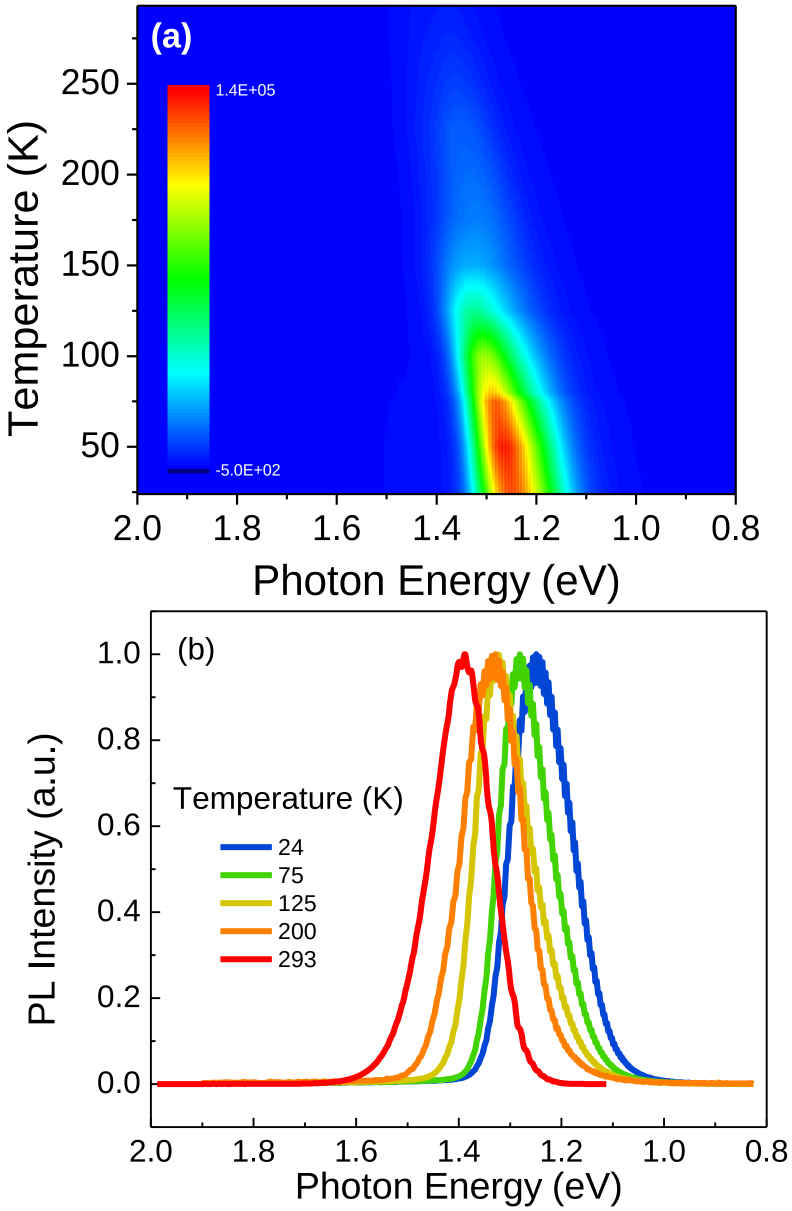

Supplementary Figure 4. (a) Photoluminescence map from the $FASnI_3$ film; the colour reflects the PL intensity. The excitation power density is 0.32 μJcm^{-2}. (b) Normalized emission spectra at selected temperatures.

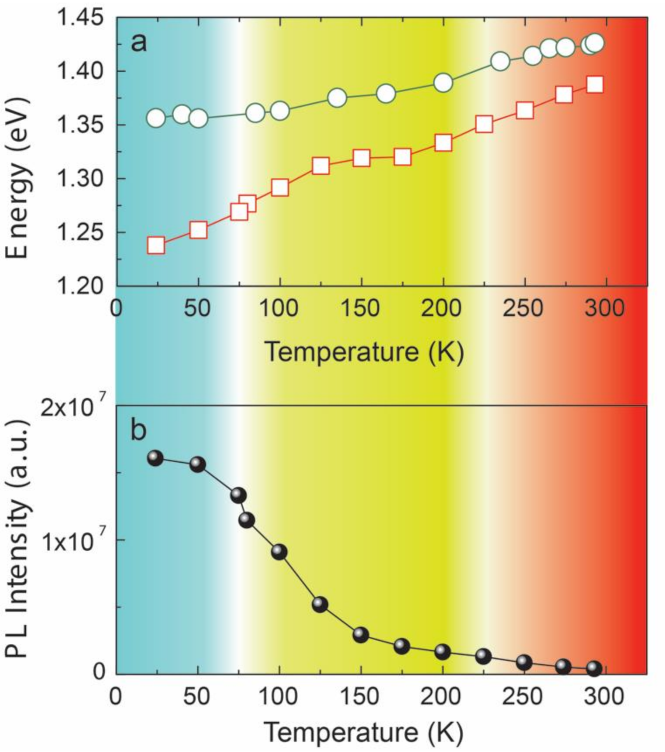

Supplementary Figure 5. (a) Comparison of absorption edge energy and emission energy as a function of temperature. (b) Temperature-dependent integrated PL intensities for the $FASnI_3$ film under excitation of 0.32 μJcm^{-2}.

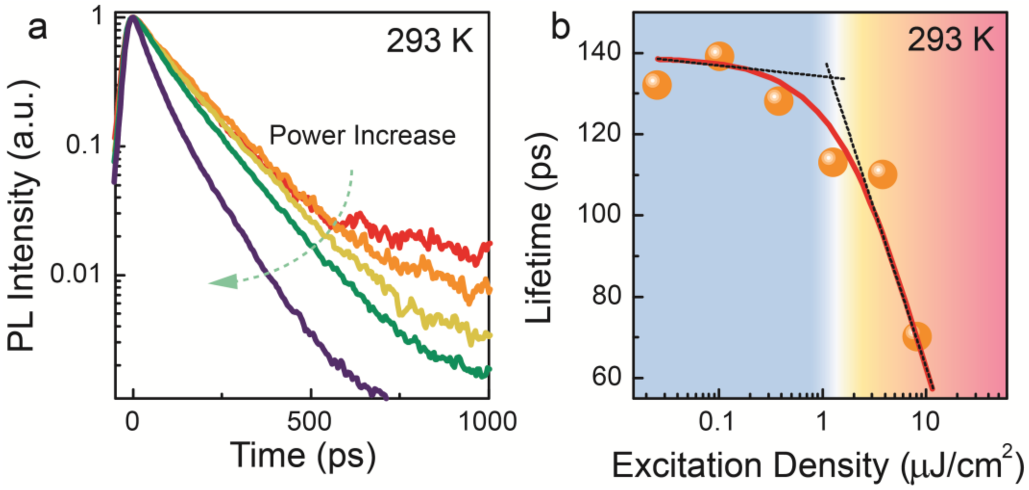

Supplementary Figure 6. (a) Normalized PL dynamics of a $FASnI_3$ thin film under different excitation power density at 293 K. (b) Excitation-intensity dependence of the effective PL lifetime film. The dashed lines are intended as a guide to the eye. The PL lifetime reduces very little under the excitation intensities below ~1.28 μJcm^{-2}, which is in the range of excitation power density where the slope of the power-dependent PL intensity is unity. This behavior implies that rather than bimolecular recombination that is strongly fluence-dependent, monomolecular recombination is heavily dominant in $FASnI_3$.

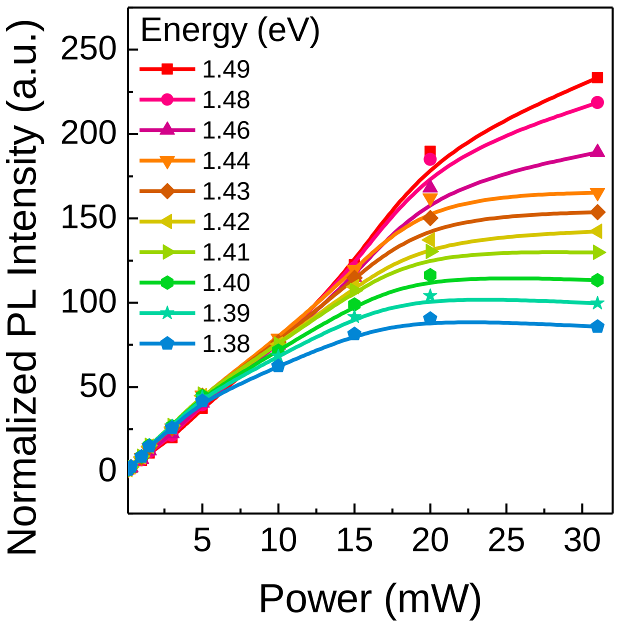

Supplementary Figure 7. Normalized PL intensity as a function of excitation power at varied energy, showing saturation of PL intensity at low energy range of the emission (1.38-1.44 eV).

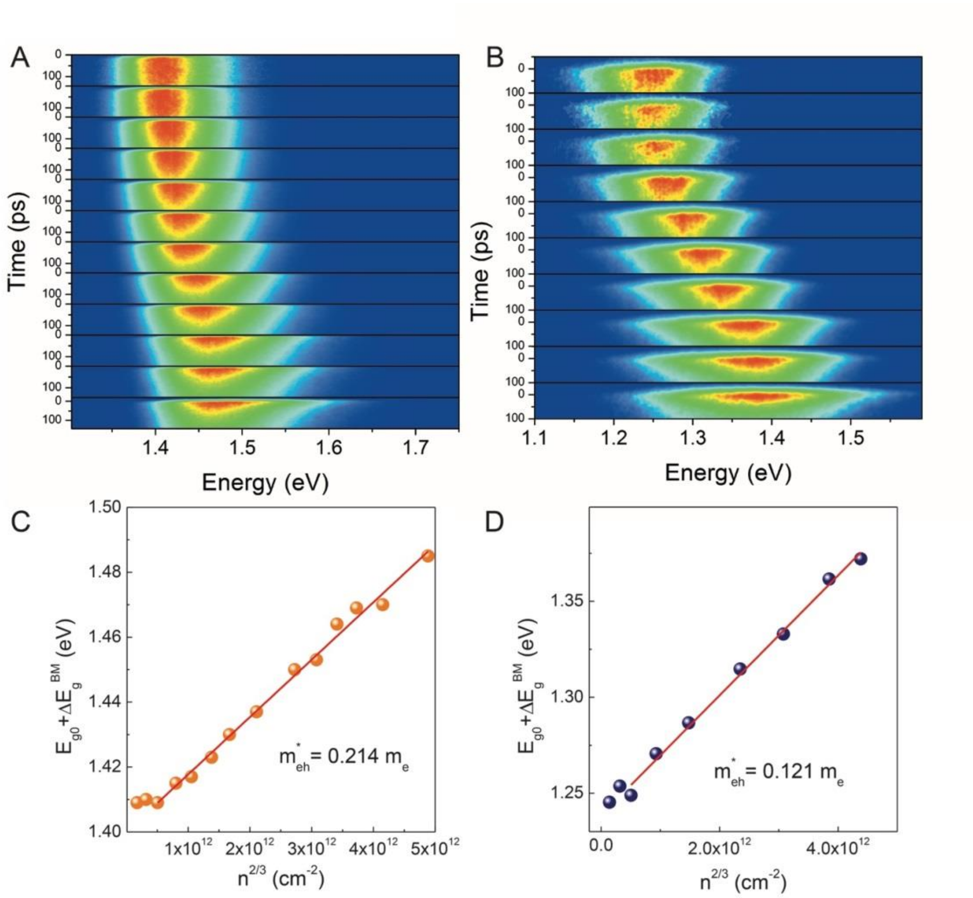

Supplementary Figure 8. Energy- and time-resolved photoluminescence spectra of perovskite thin films for varied excitation densities at 293 K (A) and 24 K (B), showing that the emission peak after excitation blue shift as excitation increases. (C) and (D), emission peak energy at around t = 0 versus the photocarrier density $n^{2/3}$ at 293 K and 24 K, respectively.

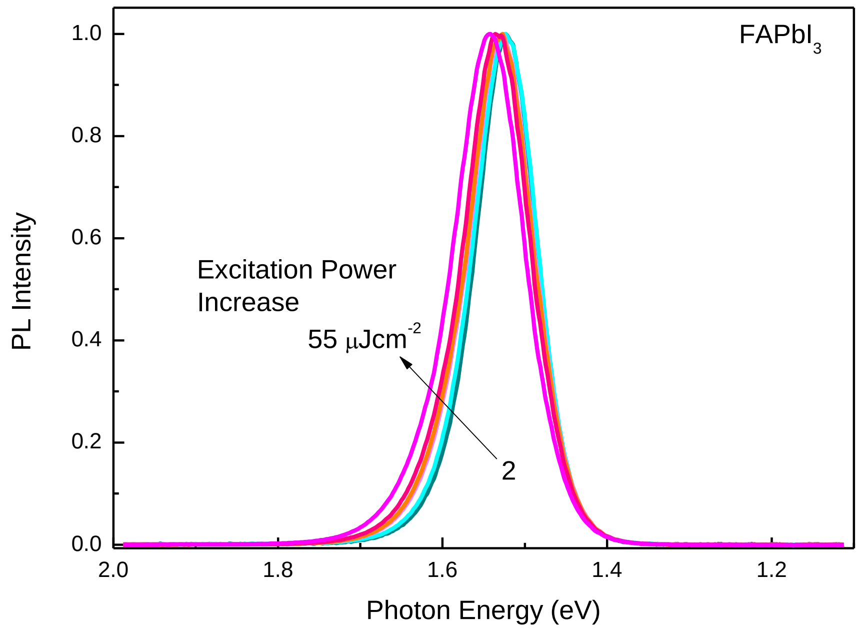

Supplementary Figure 9. Power-dependent photoluminescence spectra of the $FAPbI_3$ thin film at 293 K, showing a small shift at high excitation density.

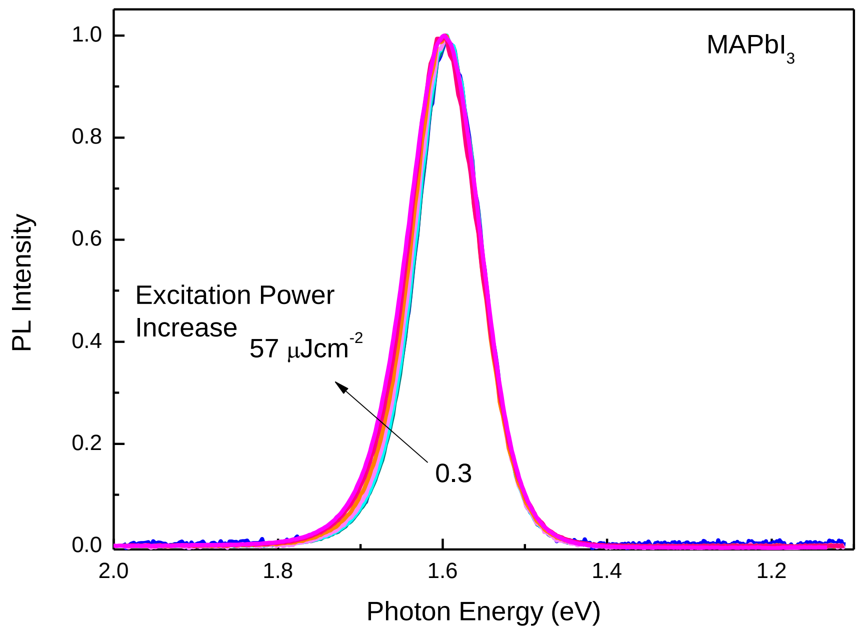

Supplementary Figure 10. Power-dependent photoluminescence spectra of $MAPbI_3$ thin film, showing that the peak energy is almost identical at varied excitation intensities.

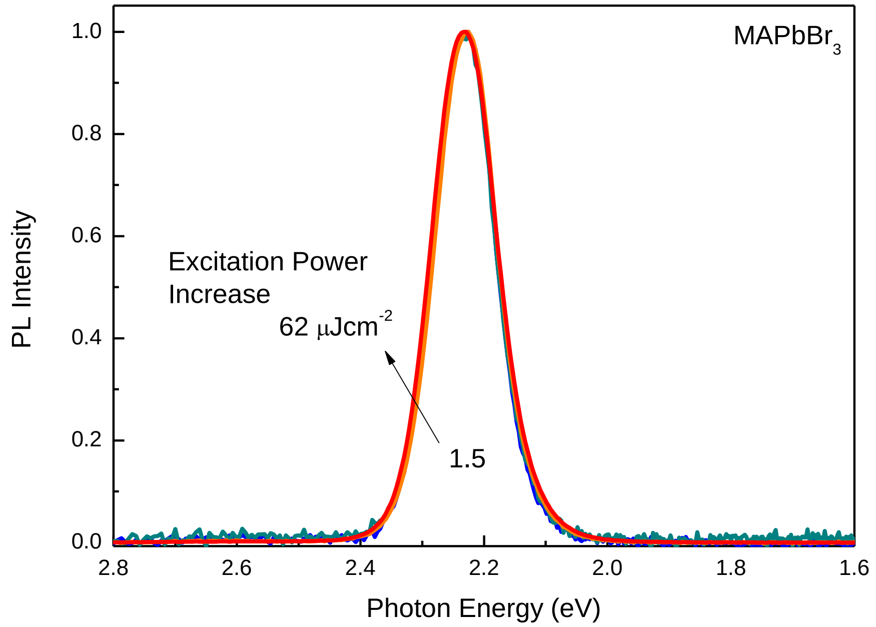

Supplementary Figure 11. Power-dependent photoluminescence spectra of $MAPbBr_3$ thin film, showing that the peak energy is identical at varied excitation intensities.

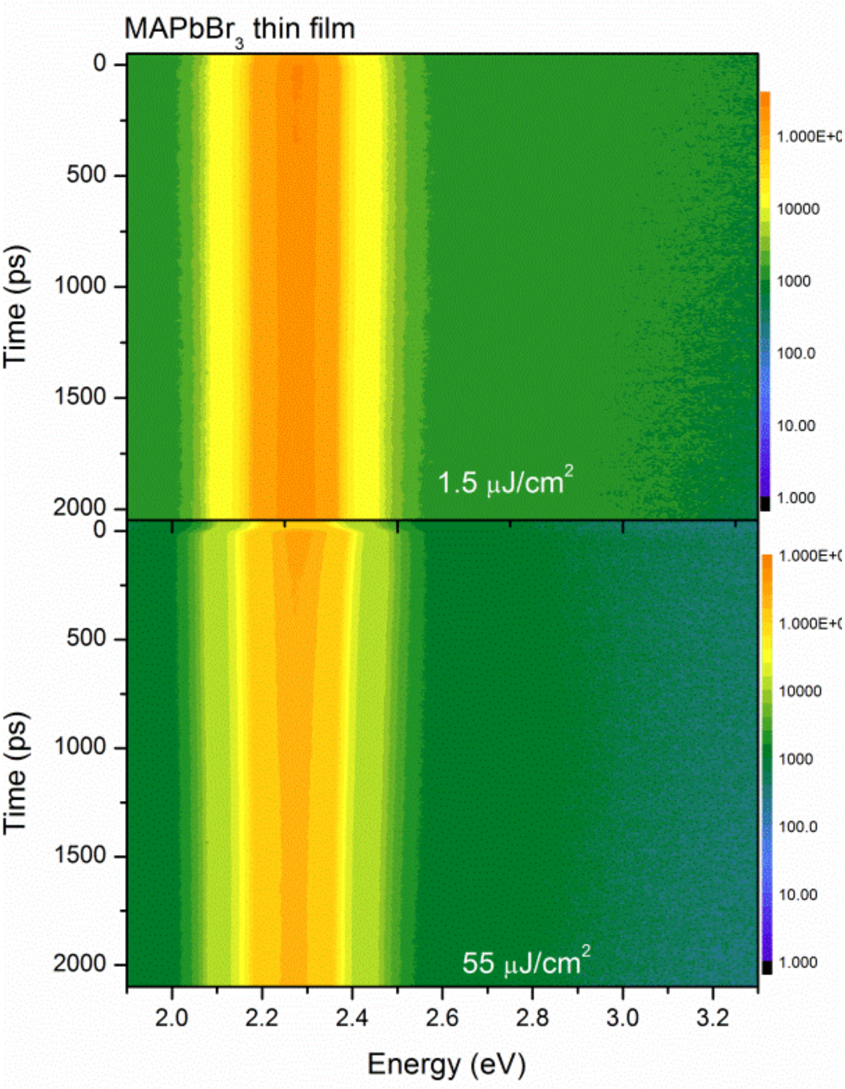

Supplementary Figure 12. Energy- and time-resolved photoluminescence spectra of perovskite thin films at low and high excitation density for $MAPbBr_3$ thin film.

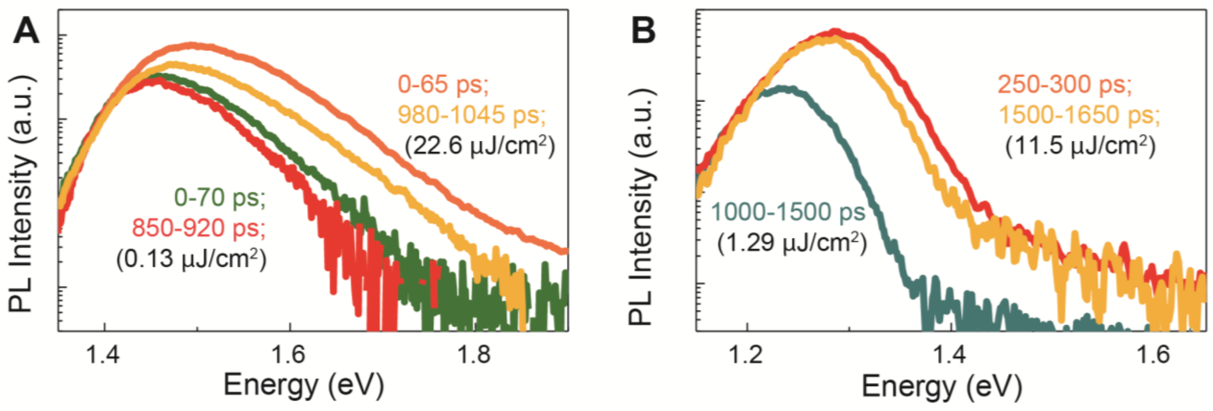

Supplementary Figure 13. Normalized PL spectra at various delays after excitation at selected excitation density for (a) 293 K and (b) 24 K, respectively.

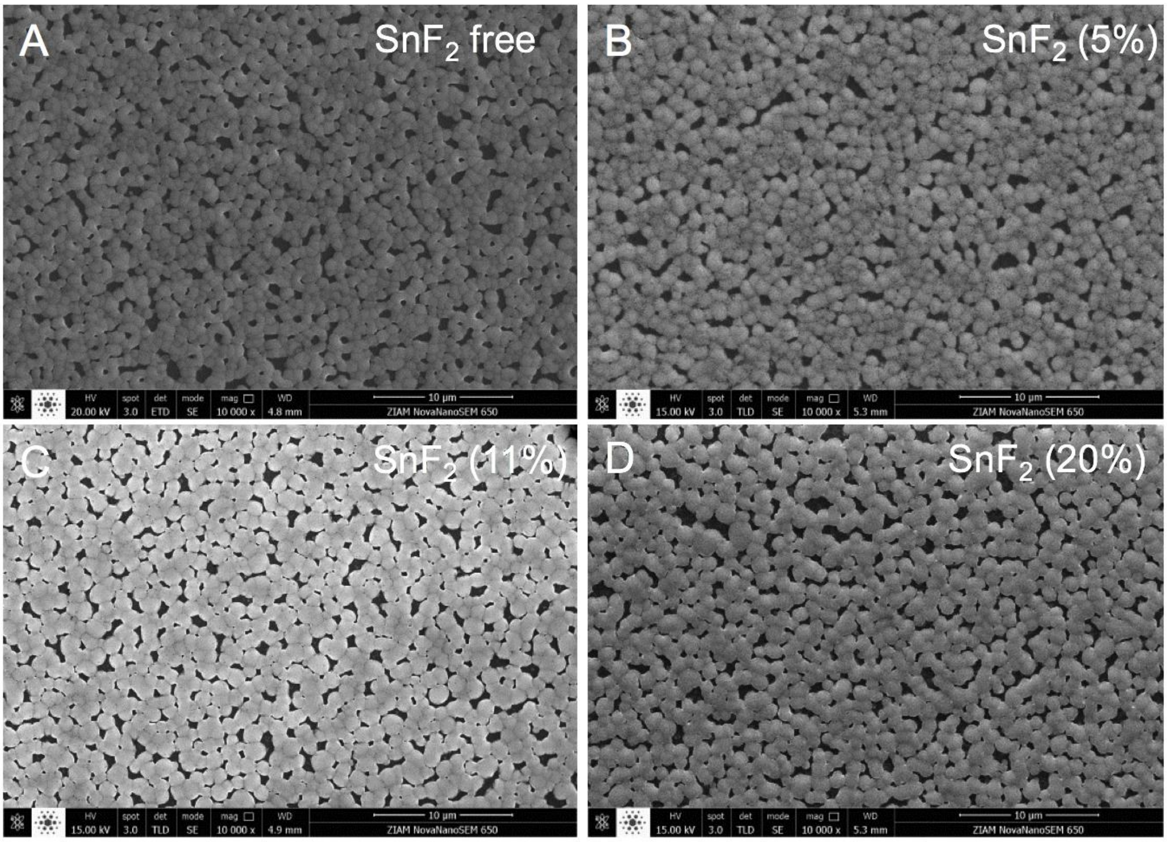

Supplementary Figure 14. SEM micrographs of FASnI3 films with different amounts of SnF_2 additive.

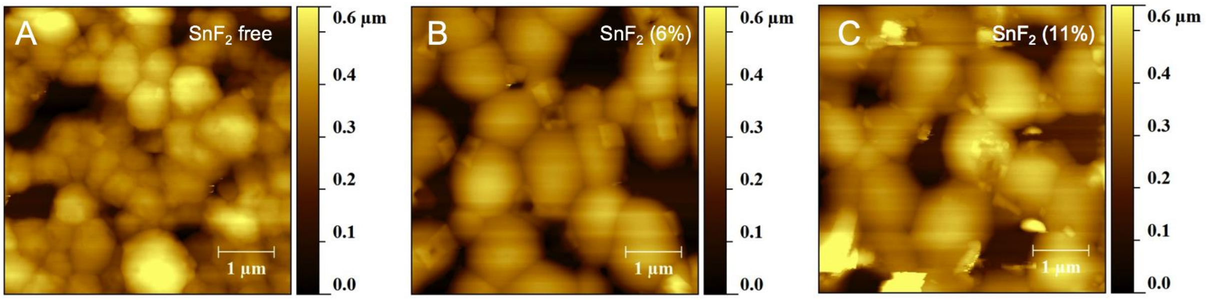

Supplementary Figure 15. AFM micrographs of SnF_2-free $FASnI_3$ films and thin films with various concentrations of SnF_2 additive.

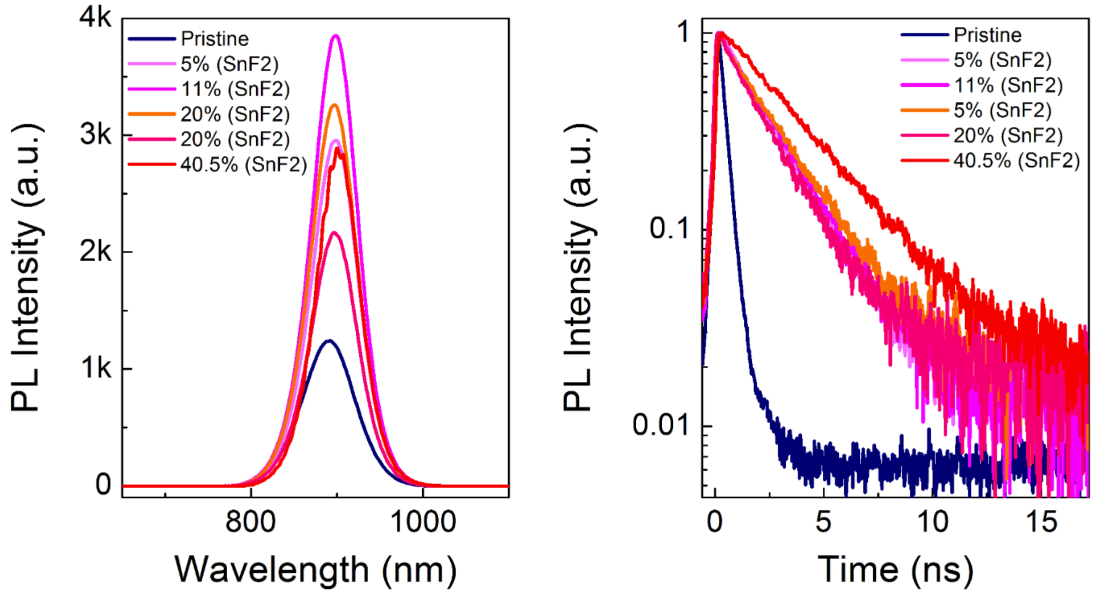

Supplementary Figure 16. (A). Photoluminescence spectra of FASnI3 thin films without SnF_2 and with various concentrations of SnF_2 additive. (B). Time-resolved photoluminescence decay spectra of SnF_2-free $FASnI_3$ film and with various concentrations of SnF_2.

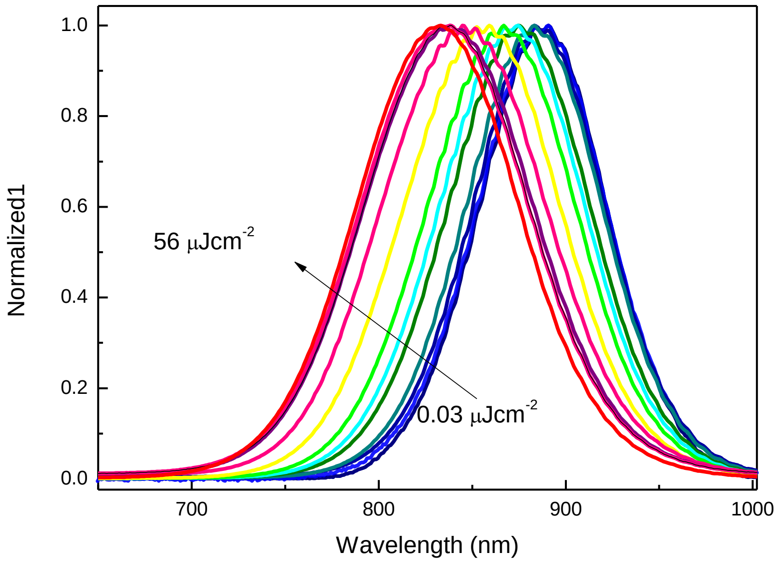

Supplementary Figure 17. Normalized PL spectra of a $FASnI_3$ thin film with addition of SnF_2 (11%) under different excitation densities.

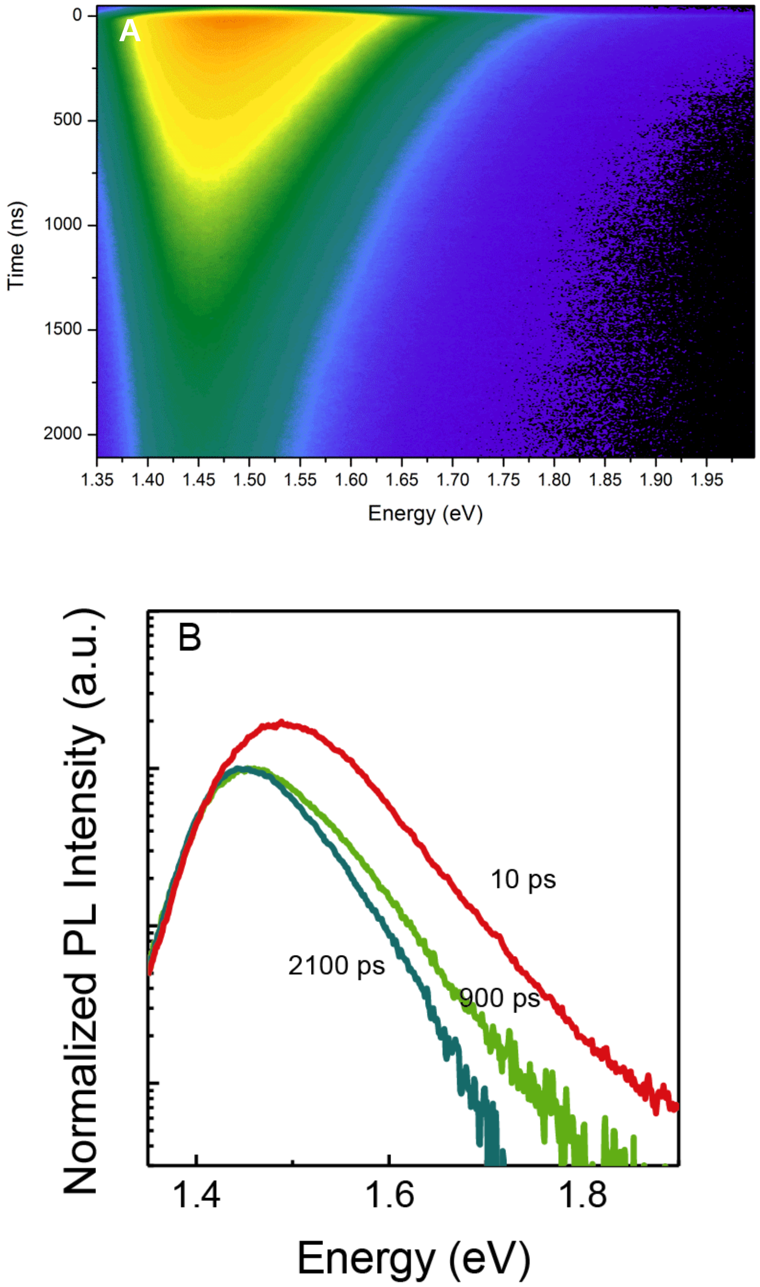

Supplementary Figure 18. (a) Energy- and time-resolved photoluminescence spectra of $FASnI_3$ (SnF_2) thin film under excitation of 61 μJcm^{-2}. (b) PL spectra for $FASnI_3$ thin film at different delay times.

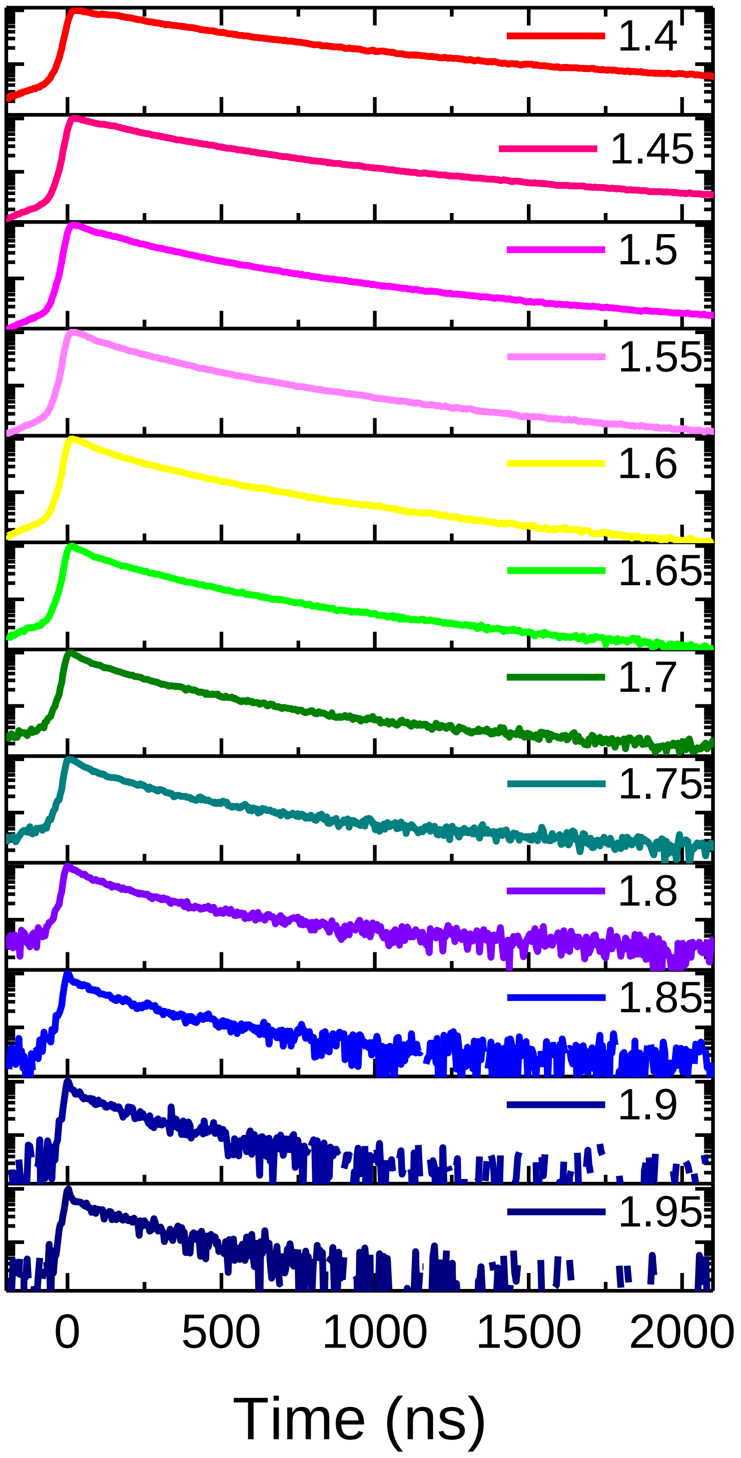
1.4
1.45
1.5
1.55
1.6
1.65
1.7
1.75
1.8
1.85
1.9
1.95
0
500
1000
1500
2000
Time (ns)

Supplementary Figure 19. Time-resolved photoluminescence at various energies in $FASnI_3$ (SnF_2) thin film under excitation of 61 μJcm^{-2}.

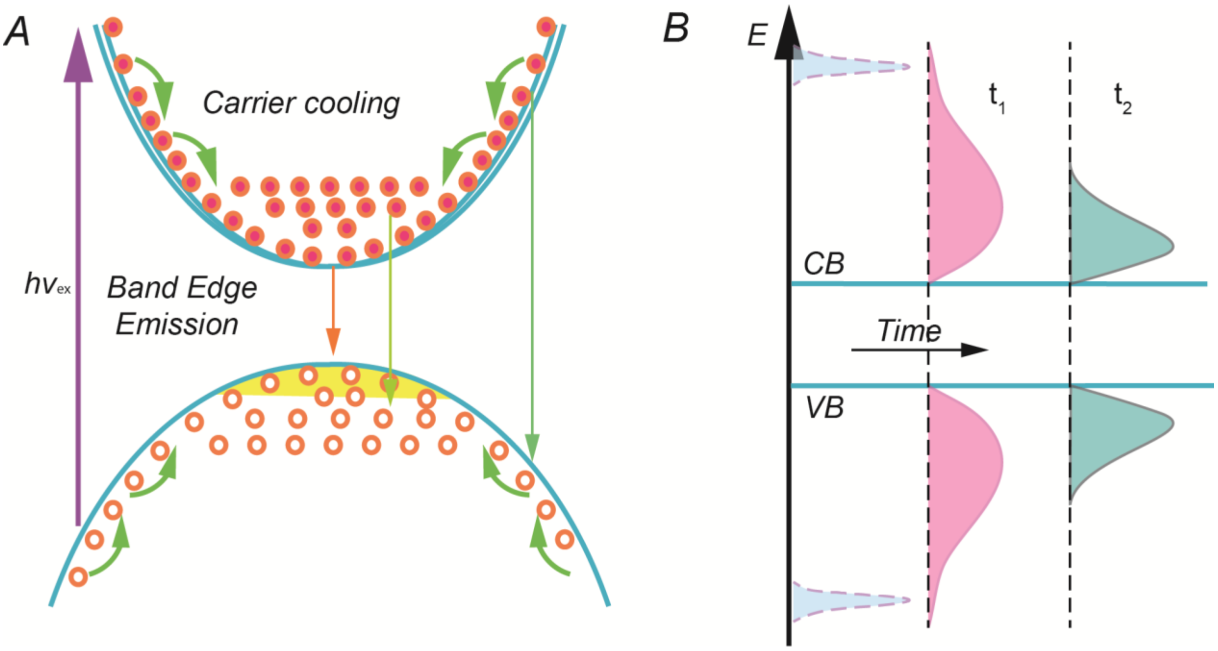

Supplementary Figure 20. Schematic of the generation, cooling, and band filling of excess carriers. Photogenerated charge carriers return to equilibrium via thermalization through carrier scattering, cooling by phonon emission.

Supplementary Note I.

Temperature dependent absorption and photoluminescence.

The temperature-dependent absorption measurements were carried out under vacuum in a cryostat from 293 K to 24 K. Supplementary Figure 2 shows the optical absorption spectra taken at 293 K and 24 K, respectively. The absorption onset at room temperature is localized at around 1.43 eV (865 nm), near to the values established for the band-gap by DFT calculations and previous measurements [2]. The extent of the absorption tail with an exponential decay of the density of states, known as the Urbach tail, is observed. Urbach energy is given by $\alpha \propto \exp[(\hbar\omega - \hbar\omega_0)/E_u]$, where α is the absorbance. The fit to the Urbach tail yields an Urbach energy of ∼32 meV that is twice the value (15 meV) for lead halide perovskites [3,4].

Supplementary Figure 2 shows the contour plot of temperature dependent absorption spectra. Upon cooling down, the absorption monotonically shifts towards lower energy reaching 1.35 eV (916 nm) at 24 K. A slight change in the variation of the optical absorption spectrum occurs at about 80 K, which is possibly in relation to one of the reported phase transitions. Two possible phase transitions at low temperature are reported: one (Phase Transition I) is at 180 K, and the second one possibly occurs at around 75 K [1,5]. Supplementary Figure 4 (a) shows temperature-dependent photoluminescence map under excitation power density of 0.32 μJcm^{-2}. The PL spectra at selected temperatures are displayed in Supplementary Figure 4 (b). The room temperature emission is centered at around 894 nm, which corresponds to a Stokes shift of ~29 nm. It is reported that the $MASnI_3$ exhibit a secondary, higher-energy absorption and emission feature near 1.75 eV [6], which is properly intrinsic to the band structure. A similar secondary onset is observed in the absorption spectra of $FASnI_3$. The higher-energy transition may occur at around 1.9 eV at 293 K, and it red shifts when temperature decreases. However, we observed no emission from this higher-energy transition. A weak secondary absorption 0.35 eV above the band gap was attributed in $MASnI_3$ to the presence of split-off states in the conduction band [6]. The presence of such a secondary optical transition at R in halide perovskites was originally presented as a possible trapping mechanism for the electrons generated in the $F_{3/2u}$ state of the conduction band [7]. The presence of an additional absorption contribution above the band gap might although be consistent with the data reported for $CsSnI_3$ thin films [8]. In $FASnI_3$ thin films, the absorption only shows a very small inflexion at about 0.4 eV above the band gap at 24 K (Supplementary Figure 2). This signature is smeared out at 293 K.

The PL peak energy, as well as the absorption onset energy, is summarized in Supplementary Figure 5 (a) as a function of temperature. The PL peak shifts continuously to low energy from 293 K to 24 K, without observation of apparent disrupt due to phase transition as commonly observed in lead-based perovskites [9,10]. The PL peak width in $FASnI_3$ thin film at 293 K is around 0.13 eV, which is rather large in comparison to their lead-based counterpart, $FAPbI_3$ (0.081 eV) but smaller than for $MASnI_3$ [6,11]. Generally, the contribution to the overall width of the PL spectrum can be ascribed to homogeneous and inhomogeneous broadening, where the former is correlated with the carrier-phonon interaction, and thus the contribution diminishes with lowering temperature, while the latter is due to scattering with ionized impurities and disorder in samples, thus an indicator of inherent structural disorder. This is further confirmed by the absorption spectra measurements, which shows a broadening of the Urbach energy. The temperature-dependent emission intensity is plotted in Supplementary Figure 5(b). The PL intensity of the $FASnI_3$ thin film is increased when the temperature reduced from 293 K to 24 K, similar to the observation in lead halide perovskites[11].

Supplementary Note II.

Power-dependent TRPL in $FASnI_3$

Supplementary Figure 6 (a) show the decay kinetics of a $FASnI_3$ thin film under different excitation intensities at 293 K. The PL lifetime reduces very little under the excitation intensities below ~1.28 μJcm^{-2}. This is consistent with the linear behavior reported in figure 1c. . It is a strong indication of monomolecular recombination in $FASnI_3$.

To unravel the carrier recombination rate associated with mono- and higher-order processes, the time-resolved PL kinetics are fitted by the simple rate equation:

$$\frac{dn}{dt} = -An - Bn^2 - Cn^3 \quad (1)$$

where *n* is the photoinduced charge carrier density, *A*, *B*, and *C* represent recombination rate constants for monomolecular, bimolecular and three-body Auger recombination, respectively.

Considering all the sets of data, the obtained parameters, A, B and C in the thin films are 7.4 × 10^9 s^{-1}, 3.4 × 10^{-9} cm^3s^{-1}, and 3.0 × 10^{-29} cm^6s^{-1}, respectively, which is consistent with the values reported for the other tin perovskites.[2,12] The large monomolecular recombination rate suggests high dopant density in $FASnI_3$ thin films. Previous Hall measurements and calculations show that the tin perovskites are p-type direct band gap semiconductors with high concentration of mobile holes resulting from spontaneous hole-doping in the crystallization process.[13–16] Here, we reasonably assume that the hole density due to unintentional doping is p_0, and that photoexcitation produces equal numbers of electrons and holes ($n = p$). Under weak excitation, the concentration of photogenerated electrons is much lower than the total concentration of holes, which are already present in the system as "background". The photoinduced electrons either recombine with the dopant holes or are captured by traps (defects or impurities). The recombination of electrons is nearly monomolecular since the additional photocarrier density is much smaller than the concentration of "background holes". If the excitation fluence is increased such that the concentration of photogenerated electrons and holes is comparable to the background holes, then the electron-hole recombination becomes bimolecular (or Auger recombination at higher excitation), resulting in a significant shortening of PL lifetime. This behavior enables us to estimate the doping density in our samples to be $p_0 \approx 4 \times 10^{17}$ cm^{-3}, extracted from the crossover point in the PL lifetime plot as a function of excitation density. This is comparable to the value reported for tin perovskites.[2,12] By recalling the bimolecular process, the radiative recombination could be rewritten as $\frac{dn}{dt} = -Bn(p + p_0)$, where $n = p$, is the photoexcited carrier density, p_0 is the ionized acceptor density and B is the radiative bimolecular recombination coefficient. We find that $Bp_0 \approx 1.4 \times 10^9$ s^{-1}, which is one-fifth of total monomolecular recombination rate (A) and much higher than that of lead-based perovskite, suggesting that radiative recombination could be active even at low charge-carrier density, which is in stark contrast with what is happening in lead-based perovskite.

Supplementary Note III.

In the band filling model, the band-edge states are filled by free carriers, resulting in a shift of the Fermi level over the conduction band (CB) (or the valence band (VB)). The shift of the Fermi level increases with increasing density, resulting in an effective blue shift of the optical band gap. It is well known that the magnitude of the shift from free-electron theory is proportional to $n^{2/3}$, which can be modeled according to the equation: [17]

$$\Delta Eg = \frac{\hbar^2}{2m_{eh}^*}(3\pi^2 n)^{2/3} \quad (2)$$

where n is the electron carrier concentration, m_{eh}^* is the reduced effective mass and is derived from the electron and hole effective masses, m_e^* and m_h^*, according to $\frac{1}{m_{eh}^*} = \frac{1}{m_e^*} + \frac{1}{m_h^*}$, and $\hbar$ is the reduced Plank constant.

To obtain a validation of the band filling interpretation, the emission peak position is plotted against the two-thirds power of the carrier concentrations ($n^{2/3}$), as shown in Figure 8 C and D. Here, we assume for $FASnI_3$ a similar effective mass for electrons and holes, and that both carriers contribute to the band filling effect. Using eq (1), a good fit of the carrier concentration dependent emission peak shift is obtained both at 293 K and 24 K. The agreement with the experimental data suggests that the band filling model captures the essential physics of these phenomena in $FASnI_3$.

Supplementary Figure 9-11 show the power-dependent PL spectra from $FAPbI_3$, $MAPbI_3$ and $MAPbBr_3$ thin films, respectively. The emission peak of $FAPbI_3$, $MAPbI_3$ shows small shift as excitation density increase. In the $MAPbBr_3$ thin film, the emission peaks are almost identical at varied excitation densities.

Supplementary Note IV.

Carrier temperature

The emission intensity is proportional to the product of the electron and hole distribution functions. The PL spectrum is given by $I_{PL} \propto \alpha(E) f_c(E_e) f_v(E_h)$, where $\alpha(E)$ is the absorption coefficient, f_c and f_v are the Fermi-Dirac distributions for the occupation of an energy level with energy E in the conduction and valence bands, respectively. At high excitation density, the photoexcited carriers are located high in the tail of the distribution function. If E_e and E_h are well above their quasi-Fermi energies and if the distributions are Maxwellian, then the product becomes [18,19]:

$$I_{PL} \propto \alpha(E) f_c(E_e) f_v(E_h) \propto \alpha(E) \exp(-\frac{E_c}{kT_e}) \exp\left(-\frac{E_v}{kT_e}\right) = \alpha(E) \exp\left(-\frac{h\nu}{kT_e}\right), \quad (3)$$

We assume here that the carrier temperature T_e is equal for electrons and holes. Then the carrier temperature can be determined by an analysis of the emission spectrum line shape. We observe that the spectra exhibit exponential high energy tails, indicating that the carrier distribution is thermalized. To extract the carrier temperature, we carried out the fitting using PL spectra at varied delay times and the absorption coefficient extracted from Supplementary Figure 2.

SUPPLEMENTARY REFERENCES

1. Mitzi, D. B. & Liang, K. Synthesis, Resistivity, and Thermal Properties of the Cubic Perovskite $NH_2CH{=}NH_2SnI_3$and Related Systems. *J. Solid State Chem.* **134,** 376–381 (1997).
2. Milot, R. L. *et al.* Radiative Monomolecular Recombination Boosts Amplified Spontaneous Emission in $HC(NH_2)_2SnI_3$ Perovskite Films. *J. Phys. Chem. Lett.* **7,** 4178–4184 (2016).
3. De Wolf, S. *et al.* Organometallic Halide Perovskites: Sharp Optical Absorption Edge and Its Relation to Photovoltaic Performance. *J. Phys. Chem. Lett.* **5,** 1035–1039 (2014).
4. Sadhanala, A. *et al.* Preparation of Single-Phase Films of $CH_3NH_3Pb(I_{1-x}Br_x)_3$ with Sharp Optical Band Edges. *J. Phys. Chem. Lett.* **5,** 2501–2505 (2014).

5. Stoumpos, C. C., Malliakas, C. D. & Kanatzidis, M. G. Semiconducting Tin and Lead Iodide Perovskites with Organic Cations: Phase Transitions, High Mobilities, and Near-Infrared Photoluminescent Properties. *Inorg. Chem.* **52,** 9019–9038 (2013).

6. Parrott, E. S. *et al.* Effect of Structural Phase Transition on Charge-Carrier Lifetimes and Defects in CH $_3$ NH $_3$ SnI $_3$ Perovskite. *J. Phys. Chem. Lett.* **7,** 1321–1326 (2016).

7. Even, J., Pedesseau, L. & Katan, C. Analysis of Multivalley and Multibandgap Absorption and Enhancement of Free Carriers Related to Exciton Screening in Hybrid Perovskites. (2014).

8. Shum, K. *et al.* Synthesis and characterization of $CsSnI_3$ thin films. *Appl. Phys. Lett.* **96,** 221903 (2010).

9. Wu, K. *et al.* Temperature-dependent excitonic photoluminescence of hybrid organometal halide perovskite films. *Phys. Chem. Chem. Phys.* **16,** 22476–22481 (2014).

10. Fang, H.-H. *et al.* Photophysics of Organic-Inorganic Hybrid Lead Iodide Perovskite Single Crystals. *Adv. Funct. Mater.* **25,** 2378–2385 (2015).

11. Fang, H.-H. *et al.* Photoexcitation dynamics in solution-processed formamidinium lead iodide perovskite thin films for solar cell applications. *Light Sci. Appl.* **5,** e16056 (2015).

12. Song, T.-B. *et al.* Importance of Reducing Vapor Atmosphere in the Fabrication of Tin-Based Perovskite Solar Cells. *J. Am. Chem. Soc.* **139,** 836–842 (2017).

13. Takahashi, Y., Hasegawa, H., Takahashi, Y. & Inabe, T. Hall mobility in tin iodide perovskite $CH_3NH_3SnI_3$: Evidence for a doped semiconductor. *J. Solid State Chem.* **205,** 39–43 (2013).

14. Kumar, M. H. *et al.* Lead-Free Halide Perovskite Solar Cells with High Photocurrents Realized Through Vacancy Modulation. *Adv. Mater.* **26,** 7122–7127 (2014).

15. Chung, I. *et al.* $CsSnI_3$: Semiconductor or Metal? High Electrical Conductivity and Strong Near-Infrared Photoluminescence from a Single Material. High Hole Mobility and Phase-Transitions. *J. Am. Chem. Soc.* **134,** 8579–8587 (2012).

16. Xu, P., Chen, S., Xiang, H.-J., Gong, X.-G. & Wei, S.-H. Influence of Defects and Synthesis Conditions on the Photovoltaic Performance of Perovskite Semiconductor CsSnI $_3$. *Chem. Mater.* **26,** 6068–6072 (2014).

17. Manser, J. S. & Kamat, P. V. Band filling with free charge carriers in organometal halide perovskites. *Nat. Photon.* **8,** 737–743 (2014).

18. Lyon, S. A. Spectroscopy of hot carriers in semiconductors. *J. Lumin.* **35,** 121–154 (1986).

19. Jang, D.-J., Olesberg, J. T., Flatté, M. E., Boggess, T. F. & Hasenberg, T. C. Hot carrier dynamics in a (GaInSb/InAs)/GaInAlAsSb superlattice multiple quantum well measured with mid-wave infrared, subpicosecond photoluminescence upconversion. *Appl. Phys. Lett.* **70,** 1125 (1997).
